# Donor polymorphisms of Rap1A rs494453 contribute to a higher risk of hepatocellular carcinoma recurrence following liver transplantation

**DOI:** 10.7150/jca.39712

**Published:** 2020-03-04

**Authors:** Rulin Zhang, Junyi Wu, Yiming Yang, Dongge Xia, Jiayong Li, Heng Quan, Ziguang Niu, Ye Yang, Jun Wu

**Affiliations:** 1 Department of Laboratory Medicine, Shanghai General Hospital, Shanghai Jiao Tong University, Shanghai, People's Republic of China; 2 Department of General Surgery, Shanghai General Hospital, Shanghai Jiao Tong University, Shanghai, People's Republic of China; 3 School of Life Science, Shanghai University, Shanghai, People's Republic of China; 4 Department of Gastroenterology, HwaMei Hospital, University of Chinese Academy of Sciences, Ningbo, People's Republic of China; 5 Department of Biochemistry and Molecular Cell Biology, Shanghai Jiao Tong University School of Medicine, Shanghai, People's Republic of China

**Keywords:** Rap1A polymorphism, hepatocellular carcinoma, recurrence, liver transplantation

## Abstract

**Background**: Hepatocellular carcinoma (HCC) recurrence appears commonly after liver transplantation (LT), and it severely affected the long-term survival of patients. Previous studies have proved that Rap1A is involved in hepatocarcinogenesis and metastasis, and demonstrated the significant association between Rap1A gene rs494453 polymorphism and HCC. However, the relationship between Rap1A rs494453 polymorphism and HCC recurrence after LT remained unclear.

**Methods**: A total of 74 HCC patients who underwent LT from July 2005 to June 2015 was analyzed. The genotypes of both donors and recipients had been confirmed as Rap1A rs494453. The independent risk factors that associated with HCC recurrence were investigated with univariate and multivariate logistic regression analysis. The recurrence-free (RFS) and overall survival (OS) were calculated with Cox regression analysis. The Rap1A rs494453 genotype frequencies were determined using the Χ² test and the minor allele frequencies (MAFs) of Rap1A rs494453 genotypes were calculated by Hardy-Weinberg equilibrium.

**Results**: We found that the donor Rap1A rs494453 polymorphism was profoundly associated with HCC recurrence after LT. Moreover, the Milan criteria, microvascular invasion and donor Rap1A rs494453 genotype were proved to be independent risk factors for HCC recurrence. Patients with donor AG/GG genotypes had a distinct lower RFS and OS than AA genotype. The TNM stage, Milan criteria, microvascular invasion, and donor Rap1A rs494453 genotype were independent factors for the RFS of LT patients.

**Conclusions**: Donor Rap1A rs494453 is a potential predictive marker for HCC recurrence risk after LT.

## Introduction

Hepatocelluar carcinoma (HCC) is one of the major cancer-related death causes 1. The main features of HCC include rapid progression, poor prognosis, and frequent tumor recurrence. Among novel therapies, the orthotopic liver transplantation (LT) is still considered the best treatment for HCC patients having cirrhosis syndrome without local or distant metastasis 2.

However, the recurrence after LT remains frequently among 1.3% to 44.9%, which depends on individual series 3,4. Abundant studies have concluded the main causes for high HCC recurrence after surgery, including microvascular invasion, poor tumor grade, larger tumor diameter, ischemia time, vascular invasion and elevated AFP 5-7. These factors are viewed to be significant prediction of HCC recurrence by famous institute, such as the Milan, University of California San Francisco (UCSF), and Hangzhou criteria 8,9.

Rap1A is a member of Ras oncogene family of small G protein, which regulates different cellular processes, including proliferation, adhesion and cancer progression. It was reported that Rap1A promoted ovarian cancer tumorigenesis and metastasis via activating ERK, MAPK, Notch pathways 10. Decreasing Rap1A suppressed cell proliferation, adhesion, and invasion in prostate cancer and several other cancer types 11-13. A recent study further demonstrated that Rap1A expression abolished the tumor-suppressive effects of EYA4 in HCC cells, which could promote the proliferation and recurrence 14. In clinical studies, Rap1A is not only overexpressed in a cohort of Oral Cavity Squamous Cell Carcinoma (OCSCC) specimens but also correlated with the clinical characteristics of the advanced tumor stage 13. As Rap1A plays important role in malignancies, it is worthy to understand the relationship between Rap1A expression and HCC recurrence after LT.

Patients who underwent LT were divided into recurrence group and nonrecurrence group for comparation, to identify the clinicopathological factors associated with the poor prognosis. Rap1A genotypes were observed to have function on HCC relapse. Hence, we endeavoured to uncover whether donor or recipient HCC susceptibility gene (Rap1A) variations contribute to the HCC recurrence after LT in a Han Chinese population.

## Materials and Methods

### Patients

We collected 74 HCC patients among Han Chinese undergoing LT from July 2005 to June 2015 at the department of liver transplantation Surgery, Shanghai General Hospital, Shanghai, China. The median age was 47.3 years (range from 32-66 years), including 65 males and 9 females. The mean follow-up period was 49.2 months (range from 2.3-120 months). The LT criteria for patients with HCC included the absence of extrahepatic malignancies, macroscopic tumor thrombosis, and extrahepatic metastasis of HCC.

Patients were treated with standard immunosuppression regimen consisting of the triple drugs combination of cyclosporin (CsA) or tacrolimus (FK506), mycophenolate (MMF) and prednisone. Lamivudine and hepatitis B immune globulin (HBIG) were used for HBV DNA positive patients to prevent hepatitis B recurrence. The antineoplastic prophylaxis of chemotherapy and targeted therapies was used for certain patients. All patients were asked to monitor tumor recurrence or metastasis by undergoing the AFP test, ultrasonography, chest X-ray, and emission computed tomography every three months for the first two years and semiannually thereafter.

### Ethics statement

Organ donation and transplantation were approved by the Institutional Review Board, Shanghai General Hospital, and carried out strictly under the guidelines of the Ethics Committee of the hospital, and Declaration of Helsinki. All patients provided written informed consent.

### Data collection

Clinicopathological data such as age, sex, hepatitis B status, cirrhosis, histologic grade (well differentiated, moderately differentiated and poorly differentiated), Milan criteria, TNM stage, tumor size, multinodular status, microvascular invasion, and pre-LT serum AFP level were recorded. The pre-transplant data were all collected within 24h before transplantation.

### DNA Isolation and Polymorphism Genotyping

LT patients were divided into two groups, patients with HCC recurrence and nonrecurrence control groups. These samples were previously stored at -80^o^C before being genotyped. DNA was isolated from EDTA-anticoagulated whole blood or liver tissues of donors and recipients using the AllPrep DNA/RNA Mini kit (Qiagen, Venlos, the Netherlands). PCR reaction system (10 ul) included 1xGC buffer, 3.0 mM Mg^2+^, 0.3 mM dNTP, 1 U HotStarTaq polymerase, 1 ul templates DNA and 1 ul PCR primer pair: 5' CAGAAGCTGGTGGAGTGGG 3' and 5' ACCTGGATAGACGCTGGCC 3'. The conditions of PCR amplification and the mass extension reaction were available on request. Genotyping was performed in a blind way. To assess the reliability of genotyping, we randomly choose more than 10% of the samples to sequence twice. Single nucleotide polymorphism (SNP) genotyping was executed using a Sequenom Mass ARRAY SNP genotyping platform (Sequenom, San Diego, CA) 15.

### Statistical analysis

Statistical analysis was performed with SPSS statistical software version 21.0 (SPSS Inc., Chicago, IL, USA). Quantitative variables were denoted as medians (±SD) and compared using Student's t-test or the Wilcoxon signed-rank test. Categorical variables were expressed as values (percentages) and compared by Pearson's Χ² test or Fisher's exact test. The allele and genotype frequencies were determined using the Χ² test. SHEsis Online Version (http://analysis.bio-x.cn/myAnalysis.php) was used to analyze Hardy-Weinberg equilibrium. Risk factors for HCC recurrence were evaluated by logistic regression analysis. Recurrence-free survival (RFS) and overall survival (OS) were calculated using Kaplan-Meier analyiss. Statistical significance was defined as *P* < 0.05.

## Results

### Patient characteristics

A total of 74 HCC patients was divided into the recurrence group (n=33, 44.6%) and the nonrecurrence group (n=41, 55.4%), as shown in Table 1, under the criteria of the disease states after LT. The median follow-up time was 26.1 months (range from 2.3 to 91 months) in the recurrence group and 71.2 months (range from 8 to 120 months) in the nonrecurrence group, and 28 deaths (37.8%) were recorded. There was significant difference between the two groups (*P*<0.01) analyzed by Student's t-test, indicating a close association of the follow-up time and OS with carcinoma recurrence. Moreover, Chi-squared test or Fisher's exact test revealed that the Milan criteria, TNM stage, tumor size, nodules number and microvascular invasion were significant risk factors that involved in recurrence (*P*<0.05). However, other factors such as age, sex, hepatitis B, cirrhosis, Child-Pugh grading, tumor differentiation, pre-LT serum AFP level, and antineoplastic prophylaxis after LT failed to influence patient recurrence events (*P* > 0.05).

### Rap1A genotype distribution and its association with HCC recurrence

The minor allele frequencies (MAFs) of Rap1A rs494453 genotypes in both donors and recipients were more than 0.01, which were calculated by Hardy-Weinberg equilibrium. When comparing the recipients and donors carring Rap1A rs494453 polymorphisms, the latter presented distinct distributions in recurrence and nonrecurrence group while the former did not show significance, as shown in Table 2. It is of note that patients who received donor Rap1A rs494453 AG/GG genotype experienced a significantly higher recurrence rate after LT than those received AA genotype (78.8% vs. 21.2%,* P*=0.001). However, the Rap1A rs494453 genotypes of recipients did not influence the incidence rates of HCC recurrence after LT.

### Risk factors for HCC recurrence after LT by logistic regression analysis

As previous studies reported that AFP level and histologic grade also reflect the recurrence rate, we included the two predictive factors into the logistic regression analysis. Univariate logistic regression analysis was carried out. We found that the Milan criteria, TNM stage, tumor size, nodules numbers, microvascular invasion and donor Rap1A rs494453 genotypes turn out to be significant determinants for HCC recurrence after LT. To avoid collinearity, the tumor size and nodule numbers were excluded and the remaining variables indeed had no collinearity (tolerance>0.1). Multivariate logistic analysis revealed that TNM stage (OR=6.444, 95% CI 2.671-26.679; *P*=0.007), microvascular invasion (OR=20.283, 95% CI 3.277-125.527; *P*=0.001), Milan criteria (OR=5.924, 95% CI 1.379-25.446; *P*=0.017) and donor Rap1A rs494453 genotypes (OR=12.014, 95% CI 2.351-61.401; *P*=0.003) were associated with HCC recurrence (Table 3).

### Association between donor rs494453 polymorphisms and survival rates

**Figure 1:**
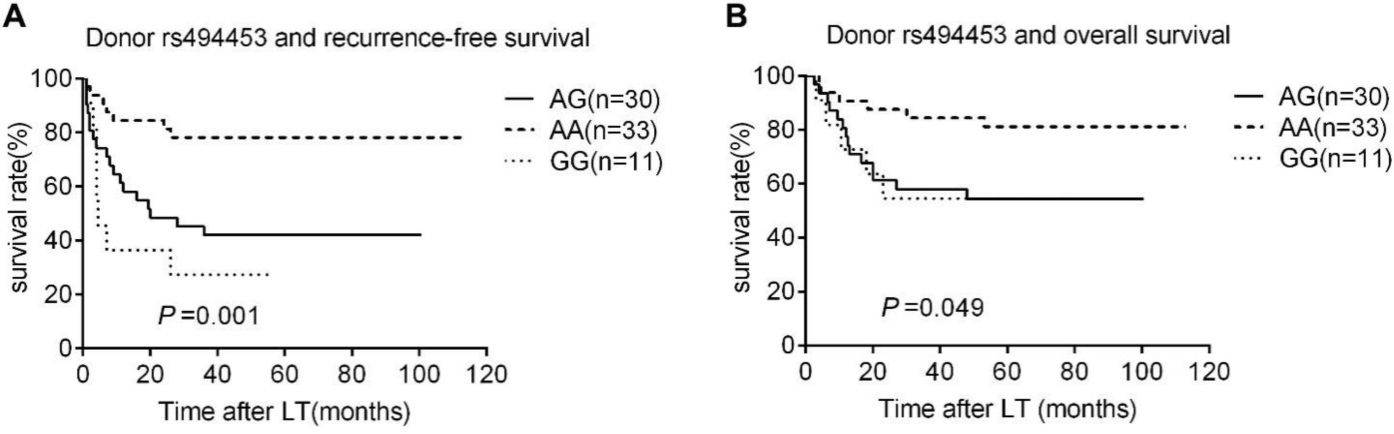
Kaplan-Meier survival estimates of recurrence-free survival (RFS) (A) and overall survival (OS) (B) among different donor genotypes (AA, GG, and AG). Patients carrying donors genotype GG and AG had the most lower RFS and OS.

**Figure 2:**
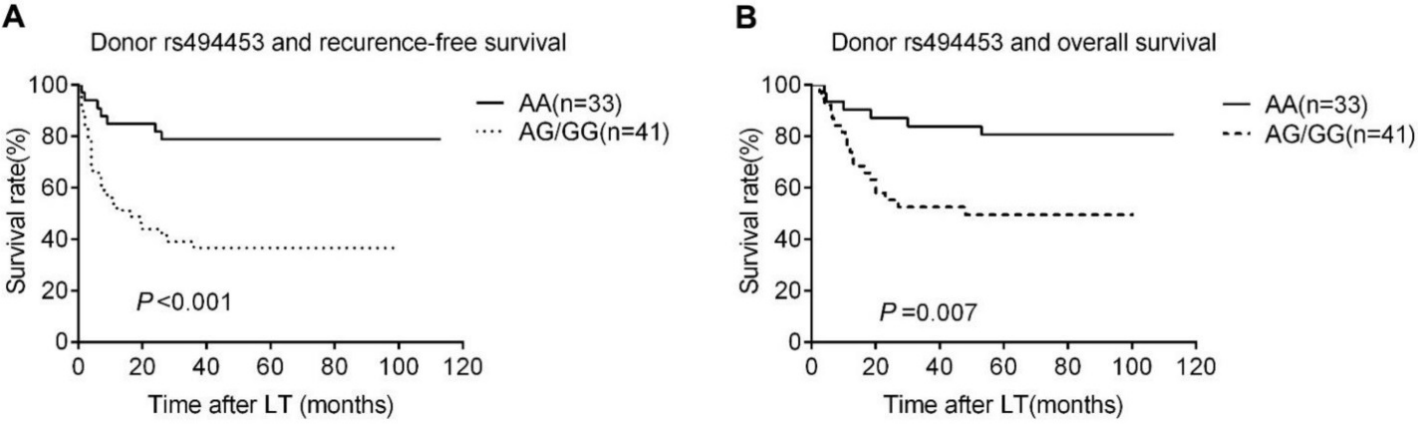
Kaplan-Meier survival estimates of recurrence-free survival (RFS) (A) and overall survival (OS) (B) between different donor genotypes (AA vs. AG/GG). Patients carrying donor homozygous AG/GG had a significantly lower RFS and OS than AA patients.

The Rap1A rs494453 genotype polymorphisms of donors, but not the recipients mainly contributed to the recurrence differences in both RFS and OS by univariate Cox regression analysis. The RFS and OS of recipients carring donor's AA, AG and GG alleles were further analysed by Kaplan-Meier survival and the log-rank test to distinguish the effect of different donor rs494453 polymorphism on prognosis. Distinctions of the AG, AA and GG genotypes of donors were observed on RFS and OS of recipients (*P*=0.001 and *P*=0.049, respectively; Fig. 1A and 1B). In addition, the RFS and OS of recipients with AG/GG genotypes of donor group were dramatically decreased compared to those with AA genotype (*P*<0.001 and *P*=0.007, respectively; Fig. 2A and 2B). However, recipient rs494453 genotypes did not show the influence on RFS and OS differentially (S1 A and B and S2 A and B).

### Risk factors for recurrence-free survival rate and overall survival rate by Cox regression analysis

We selected more impressive factors to perform multivariate Cox regression analysis, including pre-LT serum AFP level (>400ng/ml), histologic grade (poorly differentiated), TNM stage (stages 3-4), tumor size (≥5cm), number of nodules (≥3), Milan criteria (beyond), microvascular invasion and donor rs1927914 AG/GG genotype, and once again excluded tumor size and the number of nodules to prevent collinearity, as mentioned above. The remaining variables showed no collinearity (tolerance>0.1). The Milan criteria (HR=3.936, 95% CI 1.228-12.614;* P*=0.021), TNM stage (HR=16.004, 95% CI 2.047-125.110; *P*=0.008), microvascular invasion (HR=3.046, 95% CI 1.394-6.657; *P*=0.005), and donor rs494453 AG/GG genotype (HR=3.430, 95% CI 1.474-7.981; *P*=0.004) were found to be independent risk factors for RFS (Table 4). Additionally, for overall survival, the TNM stage (HR=11.490, 95% CI 1.432-92.192; *P*=0.022), microvascular invasion (HR=3.638, 95% CI 1.532-8.638; *P*=0.003), and donor rs494453 AG/GG genotype (HR=2.816, 95% CI 1.104-7.181; *P*=0.030) were also considered as independent risk factors (Table 5).

## Discussion

Rap1A is a GTPase that belongs to the Ras-associated protein (Rap) family, which is similar to Ras mostly 16, and associated with cancer initiation and progression 10. Rap1A plays a molecular switch role by converting the inactive GDP-bound state to an active GTP-bound state 17. In previous studies, Rap1A gene over-expression had been reported in various cancers, including ovarian cancer 10, breast cancer 18, and OCSCC 13. The relationship between SNP genotype and HCC recurrence after LT has been reported in VEGF gene 19, IL-15 gene 20 and HPSE gene 21, signifying that the SNP genotype of hosts, including recipients and donors, has a significant role in the progression of HCC recurrence after LT.

In this study, we explored whether the genetic variations of Rap1A were associated with the HCC recurrence after LT. Our results demonstrated that polymorphisms of donor Rap1A rs494453 were remarkably significant in the prediction of HCC recurrence after LT by Kaplan-Meyer curve analysis. Additionally, the increased risk of HCC recurrence was accompanied with AG/GG genotype of donors. Multivariate analysis showed that TNM stage, Milan criteria, microvascular invasion and the donor Rap1A rs494453 genotype AG/GG were considered as independent risk factors for HCC recurrence and RFS. In addition, the TNM stage, microvascular invasion and the donor Rap1A rs494453 genotype AG/GG were independent risk factors for OS. These results were consistent with those of previous studies 7,22,23.

However, the mechanism about how the genetic variation of Rap1A increased the recurrence risk remains unknown. Rap1A encoded protein regulates signaling pathways that affect cell proliferation and adhesion, which plays a role in tumor malignancy. MO et al. found that TNF-a activated NF-kB pathway promotes Rap1A protein over-expression then further increases the HCC progression and metastasis 14. Tao et al. found that Rap1A activates the MAPK signaling pathway to develop breast cancer 24. Other studies also showed that Rap1A promotes cell proliferation and metastasis by activating the extracellular signal-regulated kinase (ERK) and regulating the integrin-mediated cellular activities 25,26. Regarding our results, the microvascular invasion influenced mostly on HCC recurrence, which may be explanded by the circulating tumor cells from microvascular tumor thrombus 27,28.

The limitations of our study were embodied in the two respects. The first limitation was the little patient sample amount with 80.9% of it having HBV related liver disease. To obtain more reliable results, it is needed to expand the patient's sample amount and take in more HCC samples caused by different pathogenic factors, except for HBV. Besides, although we have concluded that the variant genotype influenced HCC recurrence of patients after LT surgery, there is a need to investigate the function and mechanism of Rap1A rs494453 in HCC recurrence.

In conclusion, this is the first investigation on the relationship of Rap1A gene rs494453 polymorphisms in HCC patients treated with LT. These results concluded the positive relationship between donor Rap1A rs494453 polymorphisms with HCC recurrence and OS of LT patients. Furthermore, Rap1A rs494453, microvascular invasion and Milan criteria actively predicted the risk of HCC recurrence after liver LT. These findings could be used for the clinical prognosis of liver transplantation.

## Supplementary Material

Supplementary figures.

## Figures and Tables

**Table 1:** Clinicopathological characteristics and two groups of the HCC patients

Parameter	Recurrence group (n=33)	Nonrecurrence group (n=41)	P value
**Recipient age**	47(33-64)	49(32-66)	0.689
**Recipient sex**			
Male	30(90.9%)	35(85.4%)	
Female	3(9.1%)	6(14.6%)	0.468
**Follow up time(month)**	26.1(2.3-91)	71.2(8-120)	<0.001
**Hepatitis B**			
Yes	29(87.9%)	39(95.1%)	
No	4(12.1%)	2(4.9%)	0.257
**Cirrhosis**			
Yes	24(72.7%)	35(85.4%)	
No	9(27.3%)	6(14.6%)	0.179
**Child-Pugh grading**			
A	20(60.6%)	27(65.9%)	
B+C	13(39.4%)	14(34.1%)	0.641
**Histologic grade**			
Well+moderately	27(81.8%)	37(90.2%)	
Poorly	6(18.2%)	4(9.8%)	0.292
**Milan criteria**			
Within	4(12.1%)	25(61.0%)	
Beyond	29(87.9%)	16(39.0%)	<0.001
**TNM stage**			
1-2	4(12.1%)	26(63.4%)	
3-4	29(87.9%)	15(36.6%)	<0.001
**Tumor size(cm)**			
<5	13(39.4%)	31(75.6%)	
≥5	20(60.6%)	10(24.4%)	0.002
**Number of nodules**			
<3	19(58.8%)	33(79.6%)	
≥3	14(41.2%)	8(20.4%)	0.032
**Microvascular invasion**			
Yes	19(58.8%)	3(7.3%)	
No	14(41.2%)	38(92.7%)	<0.001
**Pre-LT serum AFP level**			
≤400(ng/ml)	25(75.8%)	28(68.3%)	
>400(ng/ml)	8(24.2%)	13(31.7%)	0.479
**Antineoplastic prophylaxis after LT**			
Yes	8(24.2%)	3(7.3%)	
No	25(75.8%)	38(92.7%)	0.120

HCC: hepatocellular carcinoma; AFP: alpha-fetoprotein; LT: liver transplantation.

**Table 2:** Donor and recipient Rap1A genotype distribution and the association with HCC recurrence

	Genotype distribution, n (%)		*P* value	HWE value
	Recurrence(n=33)	Nonrecurrence(n=41)		
**Donor rs494453**				
GG	8(24.3%)	3(7.3%)		0.056
AG	18(54.5%)	12(29.3%)	0.497	
AA	7 (21.2%)	26 (63.4%)	0.003	
**Dominant model**				
AA	7(21.2%)	26(63.4%)		
AG/GG	26(78.8%)	15(36.4%)	0.001	
**Recessive model**				
GG	8(24.2%)	3(7.3%)		
AG/AA	25(75.8%)	38(92.7%)	0.201	
**Additive model**				
AG	18(54.5%)	12(29.3%)		
AA/GG	15(45.5%)	29(70.7%)	0.062	
**Recipient rs494453**				
GG	7(21.2%)	8(19.5%)		0.853
AG	22(66.7%)	27(65.9%)	0.904	
AA	4(12.1%)	6(14.6%)	0.993	
**Dominant model**				
AA	4(12.1%)	6(14.6%)		
AG/GG	29(87.9%)	35(85.4%)	0.988	
**Recessive model**				
GG	7(21.2%)	8(19.5%)		
AG/AA	26(78.8%)	33(80.5%)	0.998	
**Additive model**				
AG	22(66.7%)	27(65.9%)		
AA/GG	11(33.3%)	14(34.1%)	0.956	

HCC: hepatocellular carcinoma; HWE: Hardy-Weinberg equilibrium.

**Table 3:** Univariate and Multivariate logistic regression analysis of risk factors associated with HCC recurrence

Variables	Univariate analysis			Multivariate analysis	
	Odds ratio(95%CI)	*P* value		Odds ratio(95%CI)	*P* value
Recipient age(0 ≤50,1>50)	1.21(0.51-2.77)	0.609			
Recipient sex (0 female,1 male)	1.66(0.37-7.65)	0.421			
Hepatitis B(0 No, 1 Yes)	0.51(0.16-2.55)	0.367			
Cirrhosis(0 No,1 Yes)	0.53(0.16-1.58)	0.201			
Child-Pugh grade (0 Grade A, 1 Grade B+C)	1.21(0.52-2.77)	0.733			
Histologic grade (0 well+moderately, 1 poorly)	3.01(0.81-10.55)	0.123			
Pre-LT serum AFP level (0 ≤400ng/ml,1>400ng/ml)	0.744(0.25-2.23)	0.566			
TNM stage(0 stage1-2, 1 stage3-4)	8.33(2.38-24.88)	0.003		6.444(2.671-26.679)	0.007
Tumor size(cm) (0<5, 1 ≥5)	4.54(1.76-11.09)	0.011			
Number of nodules (0<3, 1 ≥3)	2.54(1.11-7.01)	0.042			
Microvascular invasion(0 No, 1 Yes)	14.12(4.54-47.12)	<0.001		20.283(3.277-125.527)	0.001
Milan criteria(0 within, 1 beyond)	7.44(2.32-23.01)	0.002		5.924(1.379-25.446)	0.017
Donor Rap1A rs494453 genotype (0 AA, 1 AG/GG)	2.211(1.114-5.898)	0.009		12.014(2.351-61.401)	0.003

CI: confidence interval.

**Table 4:** Cox regression analysis of the risk factors for recurrence-free survival rate

Variables	Univariate analysis		Multivariate analysis	
	Hazard ratio (95% CI)	*P* value	Hazard ratio (95% CI)	*P* value
Pre-LT serum AFP level (>400ng/ml)	0.812(0.355-1.867)	0.821		
Histologic grade (poorly)	1.621(0.668-3.930)	0.285		
TNM stage (stage3-4)	7.669(2.681-21.940)	<0.001	16.004(2.047-125.110)	0.008
Tumor size (≥5cm)	3.633(1.777-7.429)	<0.001		
Number of nodules (≥3)	2.206(1.103-4.409)	0.025		
Milan criteria (beyond)	6.754(2.363-19.301)	<0.001	3.936(1.228-12.614)	0.021
Microvascular invasion	5.950(2.919-12.129)	<0.001	3.046(1.394-6.657)	0.005
Donor rs494453 AG/GG genotype	2.596(1.034-6.515)	0.002	3.430(1.474-7.981)	0.004

HCC: hepatocellular carcinoma; AFP: alpha-fetoprotein.

**Table 5:** Cox regression analysis of the risk factors for overall survival rate

Variables	Univariate analysis		Multivariate analysis	
	Hazard ratio (95% CI)	*P* value	Hazard ratio (95% CI)	*P* value
Pre-LT serum AFP level (>400ng/ml)	1.177(0.511-2.976)	0.605		
Histologic grade (poorly)	1.209(0.443-3.23)	0.787		
Milan criteria(beyond)	4.182(1.432-12.207)	0.013		
Tumor size(≥5cm)	2.606(1.168-5.814)	0.019		
Number of nodules(≥3)	2.794(1.271-6.139)	0.011		
TNM stage(stage3-4)	23.862(3.220-176.823)	0.002	11.490(1.432-92.192)	0.022
Microvascular invasion	7.797(3.396-17.901)	<0.001	3.638(1.532-8.638)	0.003
Donor rs494453 AG/GG genotype	2.460(1.155-5.240)	0.020	2.816(1.104-7.181)	0.030

CI: confidence interval.
