# Supplementary material for: Donor polymorphisms of Rap1A rs494453 contribute to a higher risk of hepatocellular carcinoma recurrence following liver transplantation
Source: J Cancer. 2020 Mar 4;11(10):3082–8. doi: 10.7150/jca.39712 (PMC7086244; doi:10.7150/jca.39712)

Supplementary Figures

Fig.1 Kaplan-Meier survival estimates of recurrence-free survival (RFS) (A) and overall survival (OS) (B) among different recipient genotypes (AA, GG, and AG).

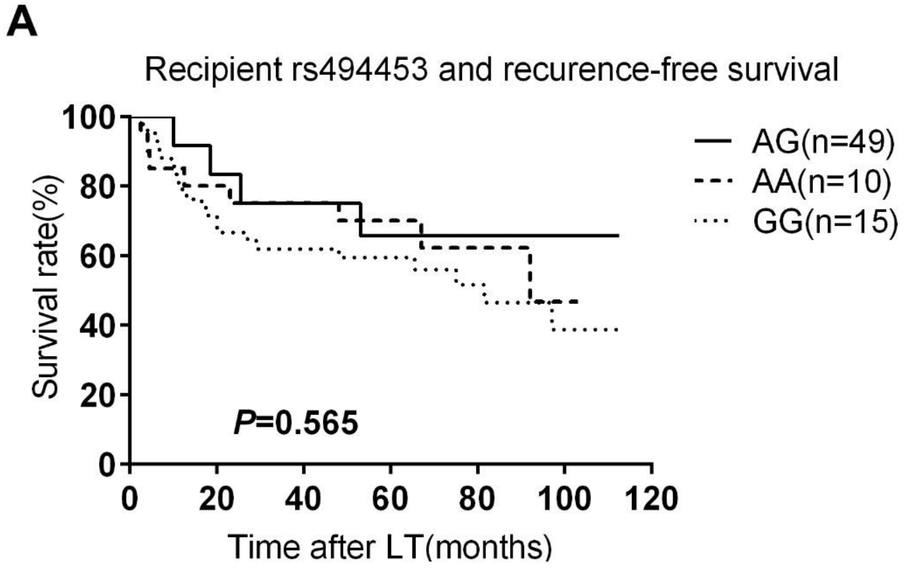

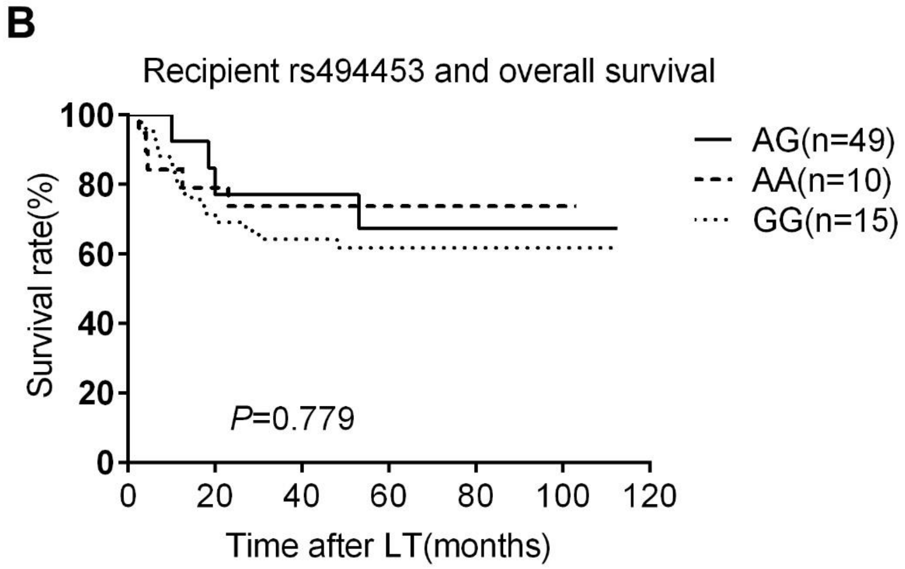

Fig.2 Kaplan-Meier survival estimates of recurrence-free survival (RFS) (A) and overall survival (OS) (B) between different recipient genotypes (AA vs. AG/GG).

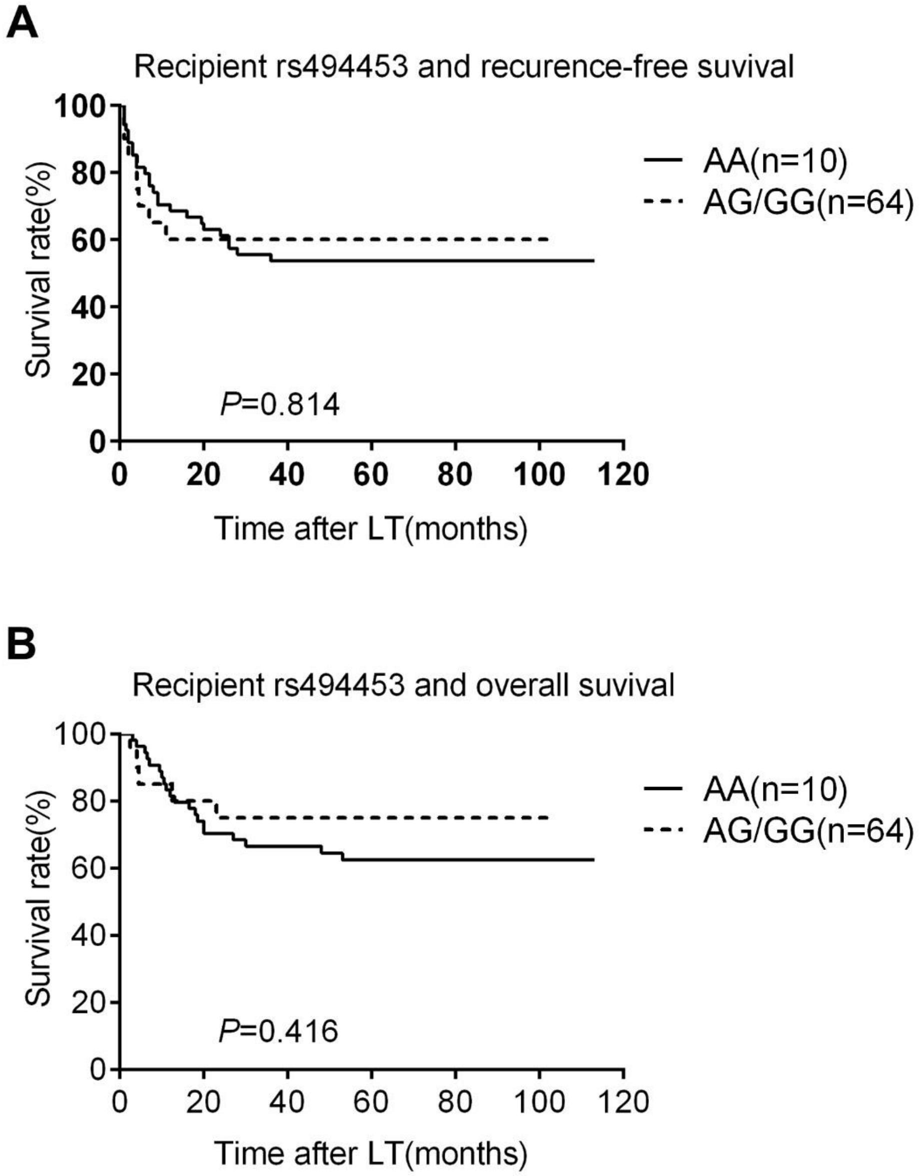

Supplement: Supplementary file 1 — Supplementary figures. [file jcav11p3082s1.pdf]
